# Management of Malignant Gastric Outlet Obstruction with Expandable Metallic Stent Placement

**DOI:** 10.4021/gr2008.11.1242

**Published:** 2008-11-20

**Authors:** Zhi Yong Wang, Li Wei Sun, Jian Liang Wu, Li Li, Ju Mei Ma, Jiao Di Hu

**Affiliations:** a Department of Digestive Diseases, the Second People’s Hospital of Hangzhou City (the Affiliated Hospital of Medical College of Hangzhou Teacher University), Hangzhou 310015, Zhejiang Province, China

**Keywords:** gastric cancer, gastric outlet, obstruction, expandable metallic stent, placement, efficacy, technique skills

## Abstract

**Background:**

To investigate the efficacy and procedural skills of metallic stent placement for malignant gastric outlet obstruction.

**Methods:**

Nine patients with malignant gastric outlet obstruction were performed metallic stent placement. Two stent placement methods were employed, the first, stents were placed under guidance of endoscopy in 7 patients (stent introducer: 140 mm in length and 4-6 mm in diameter); the second, duodenal stents were placed through endoscopic biopsy channel (3.2 or 4.2 mm in diameter) in 2 patients.

**Results:**

The stent placement succeeded at the first attempt in all 9 patients. Among the 9 patients, 3 placed with 2 x 10 cm stents, and 6 with 2 x 8 cm stents. Pylorus stents, duodenal stents, and esophageal stents were placed in 2 patients, 6 patients and 1 patient, respectively. Stents expanded about 90% confirmed by fluoroscopy 24 - 48 h after the procedure. the patients started liquid food 24 h after stent placement. The common post-procedural complications included nausea, vomiting, upper digestive tract hemorrhage and upper abdominal pain. Post-procedural obstructive jaundice occurred in 1 patient. During the 3 months follow-up, no stent migration, removal and occlusion occurred. Of the 9 patients, 3 survived 10-15 days and 2 survived 1-2 months, the rest 4 patients survived 3 months. The mean stent patency was 53.4 days.

**Conclusions:**

Expandable metallic stents placed in patients with malignant obstruction of gastric outlet effectively palliate the obstructive symptoms. Technical skills play important roles in stent placement in treatment of malignant gastric outlet obstruction, and the efficacy of metallic stent placement is associated with the location of metallic stents and therapeutic indications.

## Introduction

Gastric cancer is one of the most common malignancies, it mostly occurs in gastric antrum. The advanced gastric antral cancer often results in obstruction of the gastric pylorus, namely the gastric outlet obstruction. Though the surgical treatment may relieve the obstruction, usually the patients with advanced cancer are emaciated and in poor general condition, hence surgery is contraindicated. The gastrointestinal decompression, parenteral nutrition, and percutaneous enteral nutrition contribute little to improving the patient’s life quality. The placement of expandable metallic stent is a relatively novel approach used in recent years for relieving esophageal and gastric cardia stricture (1, 2), however, there are very limited reports on application of this approach in the management of gastric malignant outlet obstruction. In addition, the maneuvers for metallic stent placement in gastric malignant outlet obstruction are quite different from that of esophageal stent placement. The techniques of esophageal stent placement can not be adopted completely in the gastric outlet stent placement. In the past two years, we successfully performed metallic stent placement for gastric malignant outlet obstruction in 9 patients, in this report, we summarized the efficacy and our technical experiences.

## Materials and Methods

### Patients

All 9 patients were from our hospital from April 2006 to August 2007, these patients were confirmed by endoscopy and histology as malignant pylorus obstruction resulted from progressive gastric pylorus cancer, the patients’ characteristics are shown in table 1.

**Table 1:** Baseline characteristic of the 9 patients

Clinical characteristics	No. of patients
Sex (Male/female)	4/5
Age (year, mean)	46-88 (75)
Cancer sites	
Antrum	2
Antrum-pylorus invasion cancer	5
Gastric corpus, antrum and pylorus invasion cancer	2
Histology	
Adenocarcinoma	4
Gastric corpus adenosquamous carcinoma	1
Signet ring cell carcinoma	4
Stoler grade(3)	
III	1
IV	8
Extra-gastric Metastasis	4
Ascites	1

All 9 patients had one or more of the following conditions, inoperable; refusal to surgical treatment; severe deteriorating; irresponsive to short-term parenteral nutrition therapy; or with severe cardio-pulmonary diseases contradictive to laparotomy.

### Instruments

**Figure 1:**
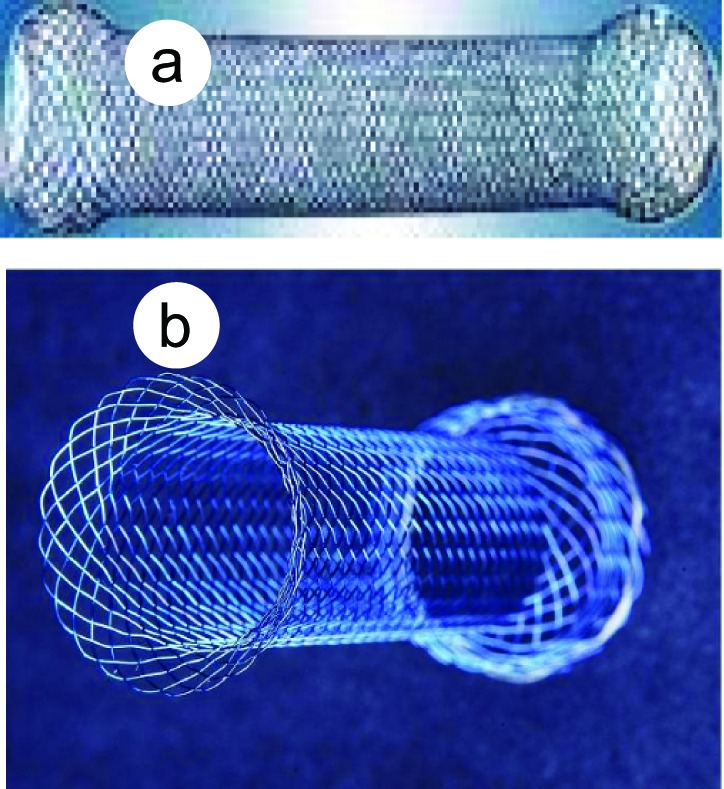
Stents used in this study. a, Duodenal stent; b, pylorus stent.

**Figure 2:**
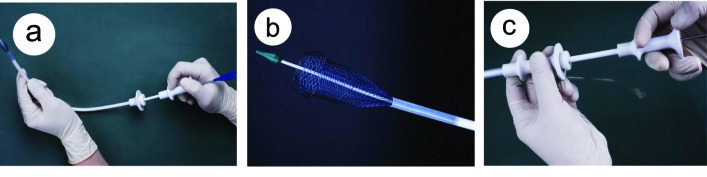
Stent delivery system. (a), stent delivery system; (b), the front tip of the delivery system; (c), the handler of the delivery system.

Olympus XQ-240 electronic endoscope was used. Stents were shape-memory MTN-shape titanium-nickel alloy intestinal stents (Micro-tech Co., Nanjing, China). Tailored design of pylorus stent was as follows, the length and width of the pylorus and antrum malignant stricture were first measured, the proximal end of the stent was designed as grand funnel-shape or plate-shpe, the distal end was designed as spherical shape (Fig. 1). The diameter of the stent was 20 - 22 mm, the stent length was 30 - 40 mm longer than the pylorus or antral stricture. The stent introducers were either, (1) dispensable luminal stent introducer MTN-CR-(4-6)/(1000-1400)-L (Micro-tech Co., Nanjing), diameter of stent introducer 4-8 mm, length 140 mm. The Savary-Gilliard dilator with a guidewire in diameter of 0.38 mm was used; (2) the duodenal stent introducer (Micro-Tech Co., Nanjing), this introducer can be inserted through the endoscope biopsy channel in diameter of with 3.2 mm or 4.2 mm. Balloon dilator with diameter of 2 cm (Fig. 2).

### Procedures

Pre-procedural preparations were as routine gastrointestinal endoscopy, the patient lied in left, endoscope was inserted and the esophagus and stomach were examined. Two apporaches of stent placement were employed.

#### (1) Stent placement under guidance of endoscopy (7 patients)

The obstructed pylorus was observed as the endoscope advanced into the gastric antrum. The endoscope was passed into the stricture and reached the middle or lower part of the descending duodenum. The length of stricture and the distance between the proximal demarcation of the stricture and the central incisor tooth were delineated. A metallic guidewire was inserted through the biopsy channel of the endoscope. The guidewire was immobilized and the endoscope was withdrawn. A introducer mounted with stent was advanced through the guidewire to the malignant stricture in gastric antrum or to the pylorus. Next, the endoscope was re-inserted below the stent introducer and rotated 90 degrees rightwards, and the stent introducer was advanced. The rotation of the endoscope made the stent introducer above clear away the gastric greater curvature, therefore the stent introducer could reach the upper part of the descending duodenum along the gastric lesser curvature. When the stent was confirmed in the appropriate location of the obstruction, then it was released.

#### (2) Duodenal stent placement through the endoscopic biopsy tunnel (2 patients)

Because our endoscope is not a therapeutic endoscopy, therefore the duodenal stent introducer can not pass through the biopsy channel. So in vitro, we first inserted a zebra guidewire throughout the biopsy channel and then into the lumen of a duodenal stent introducer mounted with stent, by doing so, the endoscope and the duodenal stent introducer were connected. The next, the guidewire was tightened, the whole assembly was inserted into the descending part of the duodenum, the zebra guidewire was pulled out, followed by withdrawal of endoscope, then the stent was released. If the stent location was inappropriate, it could be adjusted before the endoscope’s pulling out. The endoscope was only pulled out when the stent was confirmed expanded properly. In one patient, because the endoscope could not pass through the stricture, pre-dilation with a 2 cm balloon was performed, and then the stent was placed through the guidewire.

### Efficacy evaluation

The gastric outlet malignant stricture was divided into 5 grades(3), grade 0, alimentation of regular food; grade I, soft food; grade II, semiliquid food; grade III, liquid food; IV, without any food intake. The efficacy of endoscopic stent placement for pylorus obstruction was evaluated as follows, (1) complete response (CR), symptoms disappear or decrease to grade I and maintain longer than 1 month; (2) partial response (PR), stricture significantly improves and reaches grade II and may last less than 1 month; (3) minor reponse (MR), stricture improves to grade III, may last 4 weeks; (4) no response (NR): stricture does not change, stricture is at grade IV, patient deteriorates or dies.

## Results

### Efficacy of the stent placement

**Figure 3:**
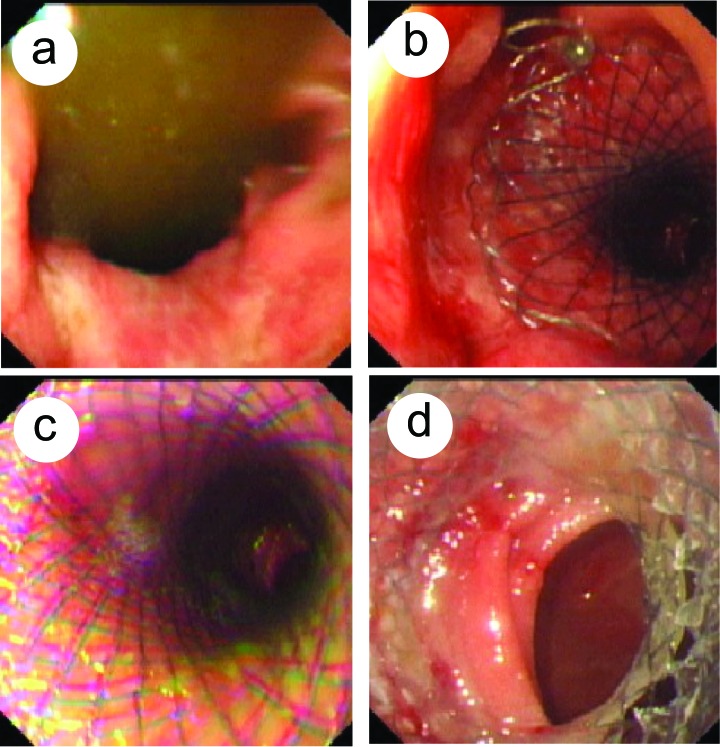
Expandable metallic stent placement for malignant gastric outlet obstruction under endoscopy in a male patient (46 years old, progressive gastric pylorus cancer). (a), stricture of the pylorus; (b), upper part of the pylorus stent; (c), middle part of the pylorus stent; (d), lower part of the pylorum stent.

Of the 9 patients, 3 patients were placed with 2 cm x 10 cm stents, 6 with 2 cm x 8 cm stents. Two patients were placed with specially designed stents, 6 patients with duodenal stents, and 1 with esophageal stent. The placed stents expanded about 90% 24 - 48 h after placement confirmed by fluoroscopy. Patients resumed food intake 24 h after stent placement. The efficacy of stent placement is shown in table 2. There were no gastrointestinal perforation and massive hemorrhage occurred. A typical patient with pylorus stent placement was shown in Figure 3.

**Table 2:** Efficacy of stents placement

Observations	No. of patients
Post-procedural obstruction	
I	1
II	3
III	4
IV	1
Efficacy	
CR	4
PR	1
MR	1
NR	3
Complications	
Vomiting, nausea	6
Upper abdominal pain	4
Melena	2
Enteric infection	1
Jaundice	1
Electrolytes disorder	1
Mean stent potency	53.4 day
Survival	
10-15 day	3
1-2 mo	2
>3 mo	4

CR, complete response; PR, partial response; MR, minor reponse; NR, no response.

### Procedural complications

Post procedural jaundice and electrolytes disorder occurred in one patient and this patient died 10 days after the procedure. Two patients with nausea and vomiting were given gastrointestinal decompression, approximately 1800 ml was drained daily, these two patients deteriorated quickly with intractable electrolytes disorder 15 days after stent placement, then the patients refused further treatment. Vomiting and nausea occurred in a patient with gastric antral cancer invaded the gastric corpus, a second stent was placed in this patient. All patients were followed up for 3 months, there were no stent migration, removal and occlusion (Table 2).

## Discussion

The incidence of gastric cancer in China is very high, a quite number of patients are in progressive stage with extra-gastric metastasis and are inoperable. In addition, some elderly patients can not be operated due to the severe cardiac-pulmonary disorders and diabetes mellitus. Therefore, in all of these patients, only the conservative treatments are considered.

For some relapsed patients post surgery, jejunostomy is sometimes performed for nutritional sustaining, these patients can not take alimentation orally, thus leading to a poor life quality for the discomfort of carrying a feeding tube. There have been reports about using memory alloy stent to treat gastrointestinal obstruction(4). Janusdhowski(5), pinto(6) and Nevitt(1) firstly reported gastric and duodenal stent placements. Scott-Mackie(7) et al modified the stent delivery system by employing stiff guidewire and specially designed stent introducer, consequently, the successful rate of internal duodenal stent placement perorally increased, this established the stent placement method for a distant gastrointestinal tract site.

Due to the long and tortuous route from mouth to duodenum, and the storage and buffering effect of stomach, when patients are aware of symptoms, the malignant gastric outlet obstruction is usually very severe; moreover, the intestinal route becomes more tortuous, all these changes make it harder to place the internal stent. In addition, the insufficient length of stent delivering system and the anatomic variation of patients make the stent placement more difficult. There have been studies on the modification of the stent delivery system in order to solve these problems, Feretis(2) et al introduced the stent with aid of a stabilizing overtube in the treatment of gastric malignant obstruction, the stent was supported as it was being advanced through the stricture, this prevented the delivery system from folding inside stomach.

In recent years, the techniques of expandable metallic stent (EMS) placement originally used in the interventional cardiovascular has been adopted in the treatment of gastroduodenal malignant obstruction, its pliable delivery system has the merits of easily passing through the tortuous gastrointestinal tract, without the disadvantages of covered or uncovered EMS.

Currently, there are two approaches for the gastrointestinal stent placement.

Firstly, stent is placed via anatomical entrance or the surgical fistula under fluoroscopy. For the complete strictures, the guidewire can not be introduced through endoscope, the thermal modalities, such as microwave or heating pole via endoscopy under guidance of fluoscopy, were used to produce a small tunnel, then the guidewire and stent were introduced. When the stent was deployed, the expansion of the stent can be adjusted through internal dilation by a balloon catheter or through changing stent location.

Secondly, stent placement under endoscopy. By this approach, the range and location of strictures can be observed precisely, therefore the appropriate size and shape of the stent can be selected. In addition, when procedural related hemorrhage, stent migration or perforation occur, prompt and proper measures can be taken under endoscopy.

In this report, we performed stent placement successfully in 9 patients with malignant gastric outlet obstruction, the patients alimentation and quality of life were significantly improved, the total efficacy rate was 66.7%, the significant efficacy was achieved in the 6 patients with gastric antrum cancer or antrum-pylorus invasion cancer. However, in two patients with gastric corpus-antrum-pylorus invasion cancer, with peritoneal metastasis and marked acites, stent placement did not yield satisfactory results. We speculated that the efficacy is closely associated with the stent location and range of lesions.

From our 9 patients reported herein, we summarized our technical experiences as follows.

Firstly, the size of stent and introducer should be selected according to the distance between the stricture and the central incisor tooth, these stents and introducers can be specially designed by the manufactures. Because the malignant stricture of the gastric outlet is distant from the incisor tooth, and the dilated proximal lumen, the guidwire and the stent delivery system tend to fold inside the stomach due to lacking of proper support, the delivery system with guidewire usually can not be advanced when reaching somewhere in the greater curvature. To overcome this difficulty, we advanced endoscope under the stent delivery system, when the endoscope reached the gastric fundus and corpus, we rotated the endoscope 90 degrees rightwards, then the stent delivery system was further advanced, due to the rotation of the endoscope, the stent delivery system can enter duodenum along the lesser curvature rather than the greater curvature.

Secondly, pre-dilation with balloon catheter was performed in severe stricture. In one patient, due to the severe stricture of the gastric outlet, the endoscope could not be passed through the stricture, and the guidewire could not be placed. In this case, we performed pre-dilation with a 2 cm balloon, then inserted the endoscope and the guidewire, finally the stent was deployed with aid of guidewire.

Thirdly, the sent release site should be lower rather than higher. The stent can be adjusted upwards easily by pulling the stent, whereas the placed stent is hard to be adjusted downwards, if this occurs, the stent must be retrieved and placed again.

Fourthly, the indications, contradictions and complications of stent placement in the malignant stricture of gastric outlet should be emphasized. In our 9 patients, the most common complications were nausea, vomiting, upper gastrointestinal hemorrhage, upper abdominal pain. Six patents complicated with post-precedural nausea and vomiting, these symptoms spontaneously relieved 2 - 3 days after stent placement, of these 6 patients, 2 patients with gastric corpus or antrum invasion cancer (one patient was 85 years old, the other was with peritoneal metastasis and abundant ascites) had significant nausea and vomiting, gastrointestinal decompression was performed and approximate 1800 ml liquid was drained daily, we speculated that this was due to the extensive lesion of gastric cancer, gastrointestinal dysfunction, gastric juice over-excretion, reduced gastric peristalsis, and loss of anti-reflux function of pylorus, all these factors lead to the reflux of intestinal contents. Therefore, we concluded that it is contradicted to stent placement in patients with gastric corpus, antrum and pylorus invasion cancer, with peritoneal cavity metastasis and ascites. In one patient, the upper abdominal pain and persistent jaundice occurred, this was because the distal end of stent suppressed the duodenal ampulla and blocked bililary drainage, in this case, the stent should be adjusted upwards, however the patient refused further interventional treatment. The suitable indications should be further studied in more patients.

We performed stent placement successfully in the first attempt in all 9 patients, we concluded that the stent placement under endoscopy is simple, direct, without exposure of X-ray, time-saving, and less traumatic, however, it needs precise localization and adequate maneuvers. Clinical data with more patients should be studied to clarify the indications and improve efficacy, and to prevent severe complications.

## References

[1] Nevitt AW, Vida F, Kozarek RA, Traverso LW, Raltz SL (1998). Expandable metallic prostheses for malignant obstructions of gastric outlet and proximal small bowel. Gastrointest Endosc.

[2] Feretis C, Benakis P, Dimopoulos C, Georgopoulos K, Milas F, Manouras A, Apostolidis N (1996). Palliation of malignant gastric outlet obstruction with self-expanding metal stents. Endoscopy.

[3] Stoller JL, Samer KJ, Toppin DI, Flores AD (1977). Carcinoma of the esophagus: a new proposal for the evaluation of treatment. Can J Surg.

[4] Dormann A, Meisner S, Verin N, Wenk Lang A (2004). Self-expanding metal stents for gastroduodenal malignancies: systematic review of their clinical effectiveness. Endoscopy.

[5] Januschowski R (1997). [Stents for the palliative treatment of malignant gastric outlet stenoses]. Dtsch Med Wochenschr.

[6] Pinto IT (1997). Malignant gastric and duodenal stenosis: palliation by peroral implantation of a self-expanding metallic stent. Cardiovasc Intervent Radiol.

[7] Scott-Mackie P, Morgan R, Farrugia M, Glynos M, Adam A (1997). The role of metallic stents in malignant duodenal obstruction. Br J Radiol.

